# Histone Deacetylase (HDAC) Inhibitors as a Novel Therapeutic Option Against Fibrotic and Inflammatory Diseases

**DOI:** 10.3390/biom14121605

**Published:** 2024-12-15

**Authors:** Maria A. Theodoropoulou, Christiana Mantzourani, George Kokotos

**Affiliations:** 1 Department of Chemistry, National and Kapodistrian University of Athens, 15771 Athens, Greece; martheod@chem.uoa.gr (M.A.T.); chrmantz@chem.uoa.gr (C.M.); 2 Center of Excellence for Drug Design and Discovery, National and Kapodistrian University of Athens, 15771 Athens, Greece

**Keywords:** fibrosis, HDACs, HDAC inhibitors, idiopathic pulmonary fibrosis, inflammation

## Abstract

Histone deacetylases (HDACs) are enzymes that play an essential role in the onset and progression of cancer. As a consequence, a variety of HDAC inhibitors (HDACis) have been developed as potent anticancer agents, several of which have been approved by the FDA for cancer treatment. However, recent accumulated research results have suggested that HDACs are also involved in several other pathophysiological conditions, such as fibrotic, inflammatory, neurodegenerative, and autoimmune diseases. Very recently, the HDAC inhibitor givinostat has been approved by the FDA for an indication beyond cancer: the treatment of Duchenne muscular dystrophy. In recent years, more and more HDACis have been developed as tools to understand the role that HDACs play in various disorders and as a novel therapeutic approach to fight various diseases other than cancer. In the present perspective article, we discuss the development and study of HDACis as anti-fibrotic and anti-inflammatory agents, covering the period from 2020–2024. We envision that the discovery of selective inhibitors targeting specific HDAC isozymes will allow the elucidation of the role of HDACs in various pathological processes and will lead to the development of promising treatments for such diseases.

## 1. Introduction

Human histone deacetylases (HDACs) are enzymes that mainly catalyze the deacetylation of lysine residues of histones, regulating chromatin structure and transcription. A large number of non-histone substrates of HDACs are also reported in the literature, highlighting the importance of HDACs in several cellular functions and conditions. HDACs can be categorized into four classes based on their sequence homology: Class I (HDAC1, HDAC2, HDAC3, HDAC8), Class IIa (HDAC4, HDAC5, HDAC7, HDAC9) and Class IIb (HDAC6, HDAC10), Class III (Sirt1–7), and Class IV (HDAC11) [1]. HDAC inhibitors (HDACis) were traditionally designed and developed as potent anticancer agents [2,3]. Several of these inhibitors are FDA-approved as treatments for cancer therapy, including vorinostat, romidepsin, belinostat, and panobinostat [4,5,6,7].

**Figure 1:**
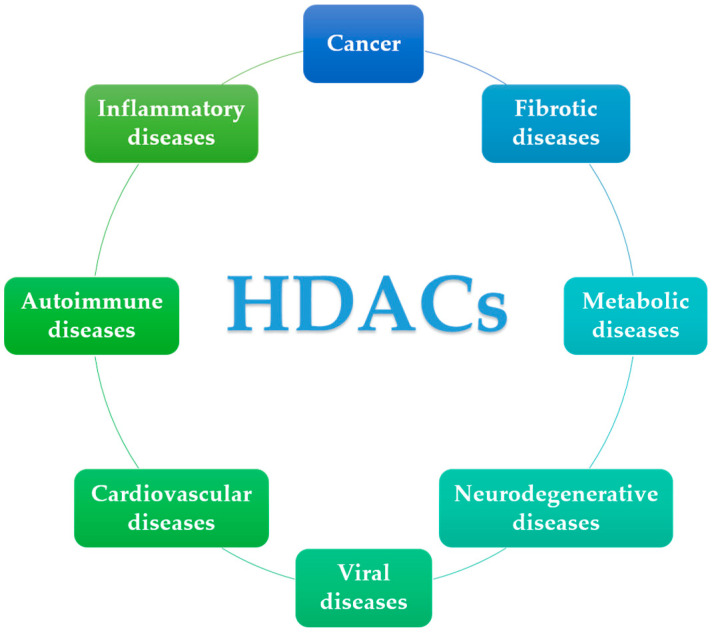
Involvement of HDACs in various diseases.

Importantly, HDAC enzymes are also involved in other diseases and disorders beyond cancer. An increasing number of reports in the literature highlight their role in neurodegenerative, cardiovascular, inflammatory, autoimmune, metabolic, and viral diseases and the development of inhibitors for the treatment thereof (Figure 1) [8]. In fact, very recently, the first HDAC inhibitor for the treatment of a disease other than cancer, has been approved by the FDA, namely givinostat for the treatment of Duchenne muscular dystrophy [9]. It is only a matter of time before other HDAC inhibitors are approved or move into clinical trials for other diseases, such as Alzheimer’s disease, pulmonary fibrosis, or inflammatory and autoimmune diseases. This perspective focuses on recent studies covering the period from 2020–2024 regarding the development and use of HDAC inhibitors as anti-fibrotic or anti-inflammatory agents.

## 2. HDAC Inhibitors in Fibrotic Diseases

Fibrosis is a common pathological process that can affect all the organs, associated with the deposition of excessive extracellular matrix (ECM) as a result of repeated tissue damage and inflammation or dysregulation of the wound-healing process, though the underlying mechanisms are yet unclear. In the last decade, new evidence suggests that epigenetic alterations, such as the acetylation of histones, play an important role in this process [10]. In fact, an upregulated expression of HDACs has been found in fibrotic organs, and several HDAC inhibitors have been reported to prevent fibrosis in organs, including the kidney [11], heart [12], and lung [13,14].

### 2.1. HDAC Inhibitors in Idiopathic Pulmonary Fibrosis (IPF)

IPF is a chronic, progressive, fibrotic interstitial lung disease of unknown etiology that occurs mainly in older adults. This orphan disease has a poor prognosis, with a high mortality rate following diagnosis [15]. Despite the fact that pirfenidone and nintedanib have been approved as treatments for IPF, they are only effective in slowing the progression of the disease without being curative [16]. Within the last decade, a number of studies have demonstrated the involvement of HDACs in fibrotic diseases [13]. Several HDACs, primarily members of Classes I and II, play important roles in the progression of IPF and have gained interest in recent years, along with the corresponding HDAC inhibitors that may constitute a new strategy for IPF treatment [13,17].

#### 2.1.1. Class I HDAC Inhibitors in IPF

**Figure 2:**
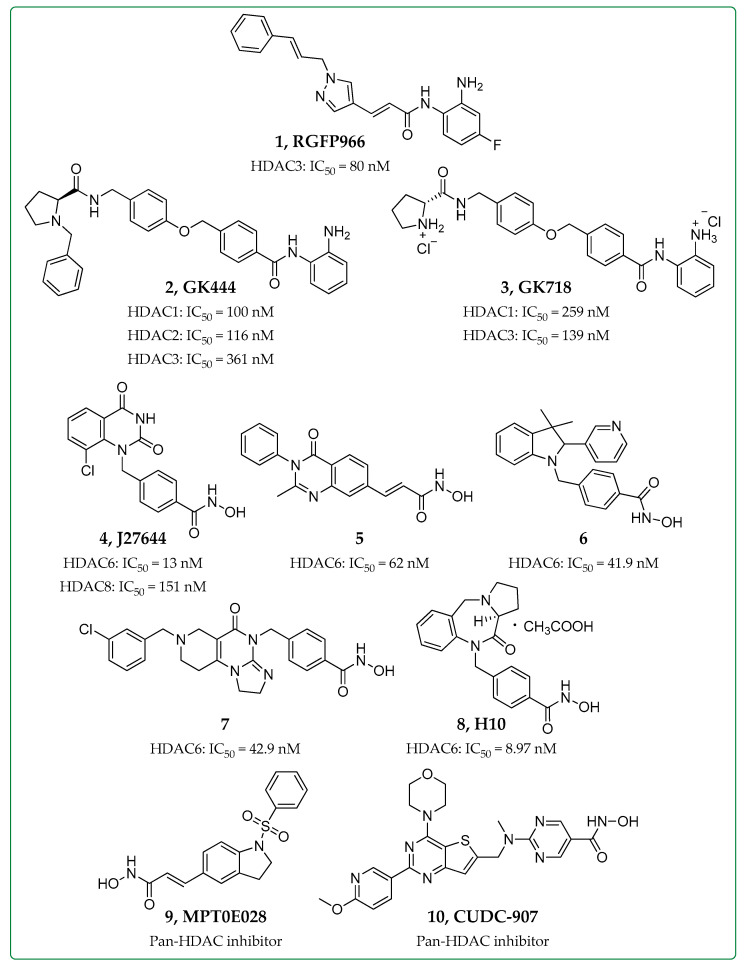
Structures and selectivity of HDAC inhibitors studied as anti-fibrotic agents in IPF [21,25,28,29,30,31].

In 2015, Korfei et al. reported that lung fibroblasts derived from patients with IPF exhibited significantly elevated expression of Class I HDAC enzymes, leading to their aberrant activation and persistence in IPF due to alterations in the chromatin acetylation status and the acetylation of various non-histone proteins [18]. In fact, HDAC1 and HDAC2 were reportedly elevated in fibrotic lesions of both IPF lung tissues and primary IPF fibroblasts. Recently, a number of studies have focused on HDAC3 with regard to pulmonary fibrosis [13]. Importantly, epithelial–mesenchymal transition (EMT) is a cellular event that leads to the conversion of epithelial cells into mesenchymal (fibroblast-like) cell types that can acquire migratory and invasive properties, playing a critical role in the development of IPF. Jeong et al. demonstrated that HDAC3 can regulate alveolar EMT markers via the AKT pathway during hypoxia and enhance the migration and invasion of fibroblasts by affecting the EMT process in a positive manner [19]. Additionally, microRNA (miR)-224 was found to be an important mediator of EMT induced by HDAC3 via downregulation of Forkhead Box A1 (FOXA1) in bleomycin-injected mice. Xiong et al. have also shown that HDAC3 was highly expressed in alveolar type 2 (AT2) epithelial cells, while lung tissues from AT2-specific HDAC3-deficient mice treated with bleomycin showed reduced fibrosis and EMT [20]. The activation of the TGF-β1/Smad3 signaling pathway was associated with the transcription of HDAC3, and the administration of RGFP966 (**1**, Figure 2), a selective inhibitor of HDAC3 (IC_50_ = 80 nM) [21], was able to protect mice from bleomycin-induced PF and EMT [20]. In 2021, it was additionally established that the upregulation of HDAC3 can result in the suppression of nuclear factor erythroid 2–related factor 2 (Nrf2), a regulator of cellular antioxidative responses, which is linked to the development of pulmonary fibrosis [22]. RGFP966 was effective in reducing the Nrf2 suppression, normalizing the fibrosis in bleomycin-induced pulmonary fibrosis mice and upregulating Nrf2 downstream antioxidant enzymes and inflammatory cytokines at doses of 10 mg/kg. On the other hand, in Nrf2 knockout mice, the effects of RGFP966 were not as prominent [23]. Toscano-Marquez et al. reported that microenvironmental signals, i.e., matrix stiffness, can promote the loss of nuclear HDAC3 and increase the expression of profibrotic genes, such as Col1a1, ACTA2, and p21 [24]. Our team recently reported the synthesis of novel *N*-(2-aminophenyl)-benzamide inhibitors of Class I HDACs, specifically HDAC1, 2 and 3, and compounds GK444 and GK718 (**2** and **3**, Figure 2, IC_50_ values in the range from 100 to 361 nΜ and 139 to 259 nM, respectively) were investigated in a mouse model of bleomycin-induced pulmonary fibrosis, showing efficacy on a preventative dosing schedule (30 mg/kg daily), with decreased Col1a1 gene expression, fibrotic masses, and collagen deposition [25]. Regarding the final member of Class I HDACs, HDAC8, limited studies have been published in the last five years with regards to IPF, though it was previously established that HDAC8 expression is increased in IPF lung tissue and NCC170, an HDAC8 inhibitor was able to attenuate pulmonary fibrosis in bleomycin-injected mice [26].

#### 2.1.2. Class II HDAC Inhibitors in IPF

In recent years, HDAC6 has been the sole member of Class II HDACs that has been widely studied for its role in IPF, and novel selective HDAC6 inhibitors have been developed and tested against models of the disease [27]. In detail, HDAC6 expression diversely affects physiological processes, such as the NLRP3 inflammasome, the EMT process, and the TGF-β-PI3K-AKT and the TGF-β-Smad signaling pathways, enhancing the inflammatory response and fibrosis of lung tissues [27]. Yu et al. developed a series of HDAC6, HDAC8, and dual 6/8 inhibitors that were first screened in TGF-β1-induced embryonic mouse fibroblasts (NIH-3T3) and hit compounds were further tested in TGF-β1-induced proliferation of human pulmonary fibroblasts (HPFs) [28]. Lead compounds J27644 (**4**, Figure 2, HDAC6/8 inhibitor, IC_50_s 13 and 151 nM, respectively) and **5** (Figure 2, HDAC6 inhibitor, IC_50_ = 62 nM) reduced α-SMA and Col1a1 protein levels and efficiently modulated fibroblast survival, countering pulmonary fibrosis. Campiani et al. developed specific HDAC6 indoline inhibitors, and among the synthesized compounds, compound **6** (Figure 2, IC_50_ = 41.9 nM) caused a reduction in proliferation and fibrotic sphere formation in organoid cultures of airway basal cells derived from IPF patients [29]. Furthermore, the same compound was able to inhibit TGF-β dependent fibrogenesis in ex vivo cultures of human lung tissues, correlated with diminished expression of ECM genes. It is worth mentioning that tumor necrosis factor-related apoptosis-inducing ligand (TRAIL) has been previously associated with the progression of fibrosis, though it has not been extensively targeted for the treatment of fibrosis. Gao et al. most recently developed a series of HDAC6 inhibitors with TRAIL-activating properties to act as multitarget agents [30]. Among these compounds, compound **7** (Figure 2, IC_50_ = 42.9 nM) showed significant antiproliferative activity in two fibroblast cell lines (NIH/3T3 and HPF) in vitro and in bleomycin-induced and silica suspension-induced mouse models in vivo, attenuating inflammation and reducing collagen deposition in the lungs of mice treated with 25 or 50 mg/kg doses via gavage [30]. Finally, the efficacy of H10 (**8**, Figure 2), a novel pyrrolo [2,1-c][1,4] benzodiazepine-3,11-dione selective HDAC6 inhibitor (IC_50_ = 97 nM), was studied in a mouse model of bleomycin-induced pulmonary fibrosis [31]. Quantitative results suggested that H10 attenuated TGF-β1-dependent fibrogenesis in lung tissue derived from mice and reduced collagen deposition at the same level as Pirfenidone, an approved anti-fibrotic drug, when both compounds were administered intraperitoneally at 100 mg/kg doses.

#### 2.1.3. Pan-HDAC Inhibitors in IPF

In 2024, MPT0E028 (**9**, Figure 2), a pan-HDAC inhibitor, was tested for its potential efficacy in bleomycin-induced pulmonary fibrosis mice [32]. Oral administration of 25–100 mg/kg of MPT0E028 reduced the fibrosis score and fibronectin, collagen, and α-SMA expression, and in human lung fibroblasts, it also induced the activation of MKP-1, inhibiting p38 and ERK phosphorylation, Smad3 and AP-1 activation, and subsequent connective tissue growth factor (CTGF) expression [32]. In addition, dual inhibitor CUDC-907 (**10**, Figure 2), targeting PI3K/AKT tyrosine kinase signaling pathway and HDACs, reportedly inhibited the phosphorylation of AKT, mTOR, Smad2/3, and promoted histone acetylation in TGF-β1-induced myofibroblasts [33]. Also, CUDC-907 attenuated collagen levels in bleomycin-induced lung fibrosis in mice receiving doses of 50 mg/kg via oral gavage, thus confirming its use as a potential treatment strategy for TGF-β1-induced lung fibrosis.

Biological evaluation in vitro and in vivo and anti-fibrotic effects of the HDACis are summarized in Table 1.

### 2.2. HDAC Inhibitors in Cardiac Fibrosis

**Figure 3:**
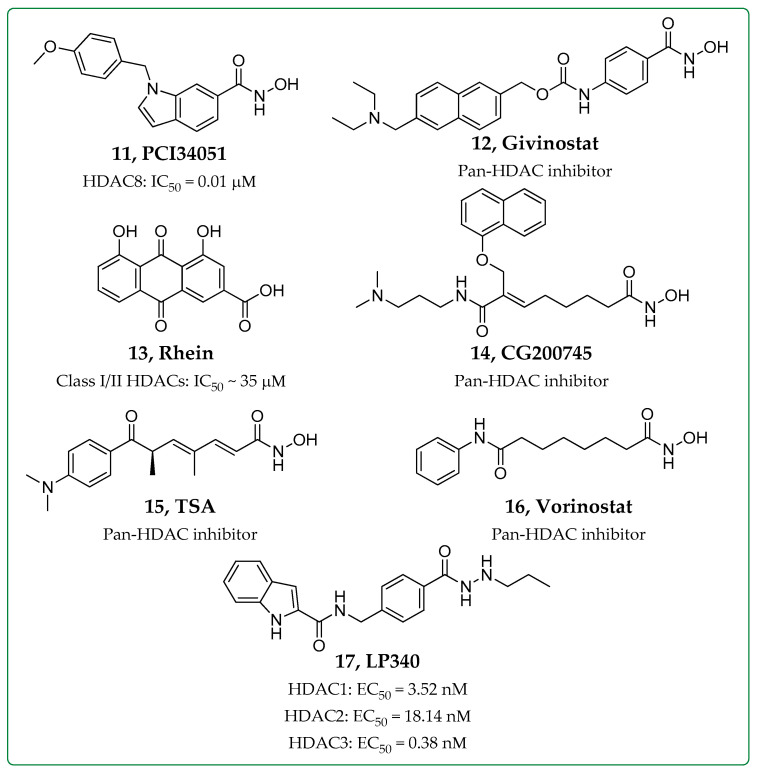
Structures and selectivity of HDAC inhibitors studied as anti-fibrotic agents in other fibrotic diseases [35,38,46].

Cardiac fibrosis, like other fibrotic diseases, is characterized by ECM deposition, and it is often the outcome of different cardiovascular diseases, such as hypertension, myocardial infarction, and cardiomyopathies. Zhao et al. have reported that knockdown of HDAC8 expression or treatment with PCI34051 (**11**, Figure 3) in rat cardiac fibroblasts can mitigate cardiac fibrosis by inhibiting the TGF-β1/Smad2/3 pathway [34]. Additionally, PCI34051 suppressed cardiac fibrosis in TAC-induced heart failure mice treated with doses of 30 mg/kg intraperitoneally. PCI34051 is an HDAC8 selective inhibitor with an IC_50_ value of 0.01 μM [35]. Further research by the same group focused on the role of HDAC8 in cardiac hypertrophy and fibrosis in an isoproterenol-induced cardiac hypertrophy mouse model [36]. Isoproterenol-infused mice treated with 30 mg/kg of PCI34051 displayed decreased cardiac hypertrophy and reduced expression of fibrosis markers (collagen type I, fibronectin, and CTGF) [36]. In addition, mass spectrometry analysis on a mouse model of diastolic dysfunction reported that treatment with givinostat (**12**, Figure 3), an FDA-approved pan-HDAC inhibitor, was able to suppress cardiac fibrosis by blocking extracellular matrix deposition and cardiac fibroblast activation [37]. Finally, Rhein, also known as cassic acid (**13**, Figure 3), an anthraquinone HDAC inhibitor of Classes I/II HDACs (IC_50_~35 μM), was investigated for its anti-fibrotic properties on fibroblast-to-myofibroblast transition of hypoxia-treated or TGF-β1-stimulated primary human ventricular cardiac fibroblasts (HCF-v) [38]. Administration of this compound dose-dependently inhibited collagen contraction, leading to increased levels of Smad7 and Smad-specific E3 ubiquitin ligase SMURF2 [38].

### 2.3. HDAC Inhibitors in Renal Fibrosis

Renal fibrosis is a common pathological manifestation of chronic kidney disease, and in recent studies, HDAC inhibitors have been shown to prevent the progression of renal fibrosis in various animal models [11]. For instance, CG200745 or ivaltinostat (**14**, Figure 3), a novel pan-HDAC inhibitor, was able to alleviate kidney fibrosis in *Col4a3−/−* mice, a murine model of Alport syndrome, and it also prevented the activation of TGF-β-Smad signaling, at an oral dose of 30 mg/kg [39]. Further, trichostatin A (TSA, **15**, Figure 3), a known pan-HDAC inhibitor, was intraperitoneally administered in a unilateral ureteral obstruction (UUO) murine model at a dose of 1 mg/kg and attenuated the accumulation of interstitial macrophages, preferentially upregulating M2c macrophages, while it also inhibited the activation of myofibroblasts and reduced the extent of fibrosis in obstructed kidneys [40]. In another study, the impact of HDAC8 inhibition by PCI34051 was investigated in vivo and in vitro on the development of renal fibrosis in a UUO murine model and in renal tubular epithelial cells [41]. Intraperitoneal administration of PCI34051, at 20 mg/kg, was able to restore the acetylation of contactin and to suppress the phosphorylation of Smad3, STAT3, β-catenin, and the expression of Snail in obstructed kidneys. It is worth noting that the maximum tolerable dose of PCI34051 reported in athymic NMRI nude mice was 40 mg/kg/day in a study regarding its effect against neuroblastoma [42].

### 2.4. HDAC Inhibitors in Liver Fibrosis

Regarding liver or hepatic fibrosis, Özel et al. published a study in 2021 regarding the in vitro effects of vorinostat (an FDA-approved pan-HDAC inhibitor, **16**, Figure 3) in LX2 cells isolated from human hepatic stellate cells (HSCs) [43]. Vorinostat treatment decreased cell viability, migration and colony formation, while it also decreased Col1a1, Col3a1, α-SMA and TGF-β gene expression levels in LX2 cells. Givinostat was evaluated for its therapeutic potential in the treatment of non-alcoholic steatohepatitis (NASH) in vivo and in vitro [44]. Liver inflammation was induced in mice through a methionine- and choline-deficient diet (MCD). Following intraperitoneal administration at a 10 mg/kg dose, givinostat was able to alleviate inflammation and reduce hepatic fibrosis. RNA-sequence analysis of liver tissues demonstrated that givinostat successfully inhibited the expression of inflammation-related genes [44]. Very recently, a hydrazide-based HDAC1,2,3 inhibitor, LP340 (**17**, Figure 3), was examined in mouse models of liver fibrosis induced by CCl_4_ treatment or bile duct ligation (BDL) [45]. LP340 inhibited HDACs 1, 2, and 3 with EC_50_ values of 3.52, 18.14, and 0.38 nM, respectively [46], while it increased the acetylation of histone-3 but not tubulin, indicating that it acts as a Class I, but not Class II HDAC inhibitor in vivo. Moreover, this inhibitor attenuated liver injury, inflammation, and fibrosis and inhibited miR23a production and the TGF-β/Smad signaling pathway in mice treated with daily doses of 0.05 mg/kg. In vitro, it demonstrated its direct inhibitory effects on immortal human hepatic stellate cells (hTERT-HSC) [45].

Anti-fibrotic activity of HDACis in vitro and in vivo in cardiac, renal, and liver fibrosis is summarized in Table 2.

## 3. HDAC Inhibitors as Anti-Inflammatory Agents

There are many small molecule drugs for the management of inflammatory diseases, including nonsteroidal anti-inflammatory drugs (NSAIDs), such as ibuprofen [47], corticosteroids, such as prednisone [48], and immunosuppressants, such as methotrexate [49,50].

**Figure 4:**
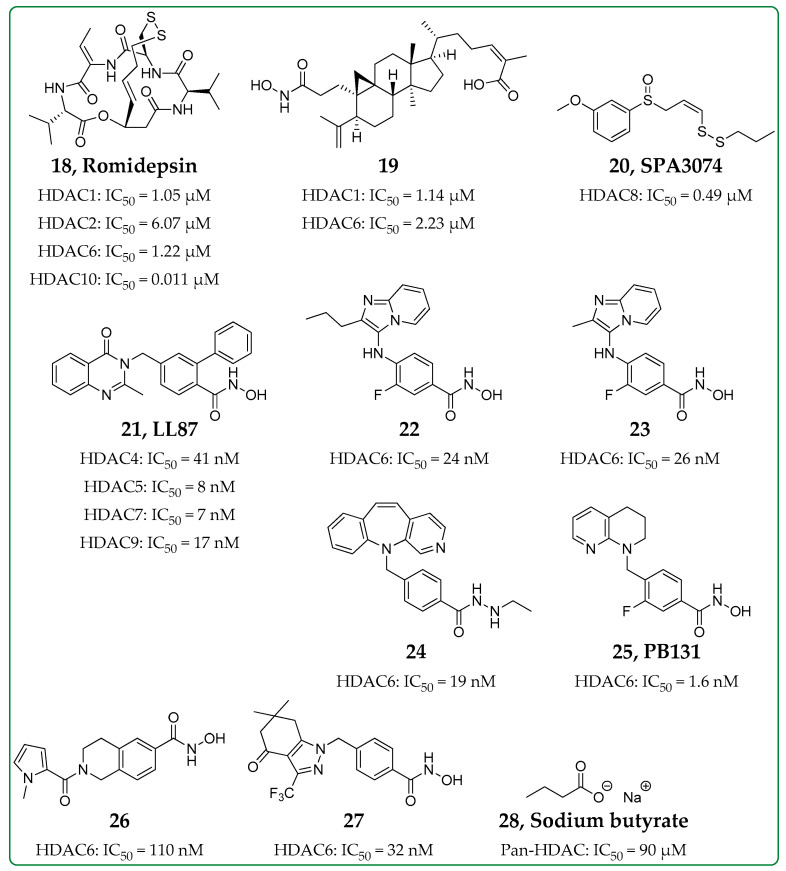
Structures and selectivity of HDAC inhibitors studied as anti-inflammatory agents [57,60,62,64,70,71,72,73,74,76].

In recent years, the role of various HDAC isozymes in inflammatory diseases has come to light. Some HDAC inhibitors, originally developed as anticancer agents, have also been studied for their anti-inflammatory properties. Vorinostat, the first pan-HDAC inhibitor that was FDA-approved, has been found to exhibit anti-inflammatory properties, downregulating the expression of inflammatory cytokines, since more than a decade ago [51,52,53]. Trichostatin A, a pan-HDAC inhibitor [54], was also shown to suppress lipopolysaccharide (LPS)-induced COX-2 expression in cell culture [55]. Romidepsin (**18**, Figure 4), an FDA-approved HDAC inhibitor, and mocetinostat, Class I selective inhibitors, were, more recently, found to exhibit anti-inflammatory properties in types of arthritis [56,57,58]. Thus, known HDAC inhibitors, established for their anticancer properties, have been further studied as anti-inflammatory agents. On the other hand, selective inhibitors of certain HDAC isozymes have been developed explicitly as anti-inflammatory agents, exhibiting low to no cytotoxicity. Although HDAC inhibition does not seem to be the most promising way to treat inflammation, in general, given the implication of HDACs in various inflammatory and autoimmune diseases [59], the development of HDACis able to simultaneously target such diseases and exhibit anti-inflammatory properties to ameliorate the symptoms of the diseases, would be beneficial.

### 3.1. HDAC Classes I and II Inhibitor

In 2021, Ni et al. synthesized a series of triterpenoid derivatives of nigranoic and manwuweizic acid and tested them for their in vitro HDAC inhibitory potency [60]. One of the most potent compounds, compound **19** (Figure 4), was able to inhibit HDAC1 and HDAC6 in low micromolar concentrations and exhibited weaker inhibition of HDAC2 and HDAC4. Compound **19** was found to successfully inhibit LDH and IL-1β production (IC_50_s 9.98 μM and 5.50 μM, respectively) while remaining not cytotoxic against J774A.1 cells (CC_50_ > 20 μM). Moreover, compound **19** was found to increase histone acetylation and inhibit IL-1β and caspase-1 expression at a cellular level, blocking NLRP3 inflammasome activation.

### 3.2. Selective HDAC8 Inhibitor

HDAC8 is also involved in inflammatory responses [61]. A selective HDAC8 inhibitor [62], SPA3074 (**20**, Figure 4), was investigated in intestinal inflammation in a mouse model of inflammatory bowel disease [63]. SPA3074 was effective against dextran sulfate sodium (DSS)-induced colitis in mice. C57BL/6N mice were treated with a daily dosage of 50 mg/kg of SPA3074. Results showed an increased SOCS1 expression in colon tissues, a protein able to suppress inflammation. SPA3074 also reduced phosphorylated Akt (p-Akt) levels and increased beneficial p-ERK1/2 and p-IκBα levels. Treatment with SPA3074 resulted in downregulated IL-13 expression.

### 3.3. Selective HDAC Class IIa Inhibitor

A highly selective and potent Class IIa inhibitor, LL87 (compound **21**, Figure 4), was studied in a collagen-induced arthritis (CIA) model [64]. The cytotoxicity of LL87 in human monocyte-derived macrophages (HMDMs) was determined, and LL87 was found to be less cytotoxic (IC_50_ > 100 μM) than other HDAC inhibitors, such as vorinostat. LL87 was studied for its in vitro anti-inflammatory properties in LPS-stimulated HMDMs, and LL87 was found to suppress the secretion of pro-inflammatory cytokines caused by LPS stimulation but was unable to inhibit TNF secretion. The role of IL-6 in upregulating Class IIa HDACs was also established. The effects of LL87 were then studied in the CIA model in rats. Dark Agouti rats were administered 10 mg/kg subcutaneously daily. LL87 was able to significantly ameliorate the arthritic symptoms in rats. The increased phosphorylation of Akt and ERK1/2 in the CIA model was also significantly reduced after treatment with LL87.

### 3.4. Selective HDAC Class IIb Inhibitors

Dysregulation of NLRP3 inflammasome activation has been related to various inflammatory or metabolic diseases [65,66]. HDAC inhibitors exhibiting anti-inflammatory effects often lead to suppression of NLRP3 inflammasome activation. HDAC6 inhibitors could act as anti-inflammatory agents, blocking NLRP inflammasome activation [67,68]. On the other hand, simultaneous inhibition of HDAC10 could potentially counteract such anti-inflammatory effects [69]. Most recently, Kraft et al. synthesized a series of relatively selective and potent HDAC6 inhibitors based on an imidazo [1,2-*α*] pyridine scaffold [70]. The most potent compounds, **22** and **23** (Figure 4), exhibited low nanomolar IC_50_ values against HDAC6. These compounds were tested in the glioblastoma U-87 MG cell line, exhibiting a weak antiproliferative activity (IC_50_s 19.5 and 16.1 μM, respectively), where their use resulted in α-tubulin hyperacetylation while having no effect on histone H3 acetylation. Compound **22** suppressed nigericin-induced IL-1β release in J774A.1 cells, though it was inactive against ATP-induced IL-1β release. Both compounds were able to suppress LPS-induced *IL1β* mRNA expression and TNF release in human THP-1 macrophages.

Another highly selective and potent inhibitor of HDAC6 was developed by Yue et al. [71] Compound **24** (Figure 4) exhibited an IC_50_ value of 19 nM against HDAC6 and over 50 times higher IC_50_ values for other HDAC isoforms, exhibiting no cytotoxicity against normal cell lines up to 100 μM. This compound also exhibited excellent oral bioavailability, as well as blood–brain barrier permeability. It was able to inhibit LPS/ATP-induced IL-1β release in vitro (IC_50_ = 2.61 μM) and in vivo, suggesting that compound **24** could act as a therapeutic agent for NLRP3-mediated diseases. C57BL/6 mice were administered 20 mg/kg of compound **24** both orally and intraperitoneally, causing a similar decrease of the serum IL-1β levels in both cases and, thus, establishing the oral activity of the compound. Moreover, compound **24** was found to be well tolerated in terms of toxicity up to 500 mg/kg. A year later, another brain-permeable selective and potent inhibitor of HDAC6 exhibiting anti-inflammatory activity, compound PB131 (**25**, Figure 4), was reported [72]. This compound was at least 100-fold more selective against HDAC6 compared with other HDAC isoforms and demonstrated no cell toxicity. PB131 suppressed IFN-γ, IL-1β, IL-2, and IL-5; however, it significantly reduced IL-10 levels, as well. Given its ability to penetrate the blood–brain barrier, this compound was studied and presented promising activity in LPS-induced neuroinflammation models, in vitro and in vivo. Intraperitoneal administration of 50 mg/kg of PB131 to C57BL/6 mice resulted in the regulation of neuroinflammation.

Brindisi et al. studied the efficacy of a selective HDAC6 inhibitor, compound **26** (Figure 4), in reducing inflammation and infection in a mouse model of cystic fibrosis [73]. This compound was not toxic and was able to reduce several interleukin and chemokine levels in a model of chronic lung infection in vivo. C57BL/6NCrl mice were treated before and after infection in the acute infection model by aerosol administration of different dosages (5, 10, or 20 mg/kg) of compound **26**, while in the chronic infection model, the same dosages were administered daily for three or seven days post-infection. Acute toxicity of these dosages was evaluated by aerosol administration of compound **26** twice. The three selected doses were well tolerated and resulted in no effects on the health status of the mice. Compound **26** exhibited no detectable toxicity in HeLa cells up to the maximum tested dose (1000 nM). It also resulted in a reduction of growth factor G-CSF and IFN-γ.

Yang et al. described the development of another selective HDAC6 inhibitor, compound **27** (Figure 4) [74]. This compound induced the acetylation of tubulin in vitro but not the acetylation of histones. Moreover, in THP-1 cells, compound **27** led to reduced secretion of TNF-α. This compound was also tested in vivo in adjuvant-induced arthritis (AIA) and CIA models, inducing improvement of arthritic symptoms in both cases. More specifically, 50 mg/kg of compound **27** was administered to Lewis rats (AIA model) orally, while DBA1J mice (CIA model) were subcutaneously treated with 15 and 30 mg/kg daily. Interestingly, combination treatment with methotrexate (1 mg/kg), used for the treatment of rheumatoid arthritis, exhibited a synergistic effect that resulted in further amelioration of arthritic symptoms. Another HDAC inhibitor that has been used in combination with methotrexate is vorinostat [75]. In the case of primary central nervous system lymphoma, the standard treatment is chemoradiotherapy employing high doses of methotrexate. In this study, SCID Beige mice (subcutaneous xenograft model) and BALB/c-nu/nu mice (intracranial xenograft model) were treated with a combination of methotrexate (50 mg/kg) and vorinostat (50 mg/kg) intraperitoneally. Notably, combinational therapy was more effective against tumors than methotrexate alone or vorinostat alone. These results are indicative of a synergistic effect of HDAC inhibitors with methotrexate in the cases of both rheumatoid arthritis and lymphoma.

### 3.5. Pan-HDAC Inhibitor

Sodium butyrate (**28**, Figure 4), a known pan-HDAC inhibitor [76], has been shown to ameliorate inflammation in allergic asthma by inhibiting HDAC1 [77]. An ovalbumin-induced mouse model was used to assess the pathways involved in this anti-inflammatory activity. Elevated levels of HDAC1, LDH, IL-5, and GATA-3 expression, HIF-1α and VEGF-α expression and phosphorylated PI3K (p-PI3K) and Akt (p-Akt) were observed in ovalbumin-induced asthmatic group. Intranasal treatment with sodium butyrate (50 mg/kg) of Balb/C mice was shown to suppress the aforementioned levels, indicating HDAC1 inhibition via the PI3K/Akt/HIF-1α/VEGF axis. Sodium butyrate was also studied in models of non-alcoholic steatohepatitis and atherosclerosis, exhibiting anti-inflammatory effects [78,79]. In non-alcoholic steatohepatitis, sodium butyrate promotes pro-inflammatory macrophage apoptosis and suppresses pro-inflammatory gene expression and secretion of cytokines [78]. In the atherosclerosis inflammation model, suppression of the inflammatory vascular intimal macrophage (Mψ) M1 and enhancement of the anti-inflammatory M2 Mψ was observed after treatment with sodium butyrate in vivo. C57BL/6J mice were fed with sodium butyrate by gavage (200 mg/kg) for two weeks. Pro-inflammatory cytokines, such as IL-1β, were also decreased, while anti-inflammatory IL-10 was increased after sodium butyrate treatment in the same model. The same was observed for cytokines mRNA levels [79]. Sodium butyrate exhibits weak cytotoxicity against cell lines ranging from 5 to 15 mM [76].

Anti-inflammatory effects of HDACis in vitro and in vivo are summarized in Table 3.

## 4. Challenges in the Application of HDAC Inhibitors for the Treatment of Fibrotic and Inflammatory Diseases

The FDA-approved HDACis, as well as other pan-HDAC inhibitors in general, have been shown to cause numerous adverse effects, including thrombocytopenia, myelosuppression, gastrointestinal problems, and cardiac abnormalities [80]. Such effects could potentially be caused due to the broad-spectrum inhibitory profiles of such compounds against HDACs in both Classes I and II. To that end, knockout phenotype studies have indicated that the deletion of Class II isoforms can result in fewer undesirable side effects compared with the deletion of Class I isoforms [81]. Therefore, severe adverse effects and cytotoxicity related to HDAC inhibition could primarily be linked to the inhibition of class I HDACs, justifying the need for isoform-selective or class-selective HDAC inhibitors. The development of specific HDACis is expected to improve treatment specificity, reducing the adverse effects.

On this axis, the majority of HDACis developed and studied for the treatment of inflammatory diseases in the last few years are Class II inhibitors, and mainly HDAC6 inhibitors. This is indicative of the tendency towards the development of isozyme-selective inhibitors, while targeting solely Class II HDACs presents less severe side effects, as shown in these studies.

Special attention has to be paid to the dosage of HDACis needed to achieve a therapeutic effect, ameliorating the symptoms of these diseases. For cancer therapy, the FDA-approved drugs are used at the following doses: vorinostat at 400 mg/dose, panobinostat at 20 mg/dose, belinostat at 1000 mg/m^2^, and romidepsin at 14 mg/m^2^ [4,5,6,7]. In the aforementioned cases where models of inflammatory diseases were studied, the in vivo administered dosages of HDACis would result in human equivalent doses ranging from 24 to 244 mg/dose [82], with the exception of the pan-HDAC inhibitor sodium butyrate. The dose administered for the manifestation of anti-inflammatory effects seems to be lower than that for cancer therapy employing pan-HDAC inhibitors vorinostat and belinostat but significantly higher than that of romidepsin and panobinostat in most cases. Certainly, it is important to take into consideration that specific dosages also depend on the way of administration of each inhibitor. Notably, the majority of HDACis studied for their anti-inflammatory properties in the literature are often administered at impractically high doses that cannot be easily implemented in human delivery due to potential unwanted adverse effects. As a result, at the moment, HDACis cannot meet expectations to replace current anti-inflammatory medications, however the simultaneous treatment of inflammatory responses in fibrotic or other disorders, could be beneficial.

## 5. Conclusions

Extensive research efforts during the last thirty years have unveiled the role that HDACs play in cancer initiation and progression [3], and as a consequence, a number of HDAC inhibitors have been approved for cancer treatment. However, although the importance of epigenetic abnormalities and post-translational modifications of histones in various diseases beyond cancer is increasingly being recognized, the role of HDACs in such diseases urgently requires further investigation. Currently, the development of effective therapeutic options against fibrotic diseases, especially against IPF, is an unmet need. The poor prognosis and high mortality rate following diagnosis with IPF highlights the urgent need for the development of therapeutic agents [83].

Selective HDACis have already been employed as useful tools to establish the involvement of specific pathways and their association with elevated expression of HDAC isozymes in the development of IPF. Inhibitors of HDAC3 were shown to upregulate the suppressed Nrf2 expression, while other Class I and II HDACis were found to suppress TGF-β-dependent fibrogenesis and Col1a1 gene expression, reducing collagen deposition and formation of fibrotic masses.

Fibrotic diseases are often accompanied by inflammation, and HDACis have been previously tested as anti-inflammatory agents. HDAC6 and HDAC8 inhibition was found to suppress pro-inflammatory cytokines and NLRP3 inflammasome activation. However, HDAC inhibition could sometimes lead to unwanted off-target effects, such as the suppression of anti-inflammatory cytokines.

The development of selective inhibitors of HDAC isozymes could lead to the elucidation of the role of each isozyme in pathological processes related to different diseases and to pharmacological agents deprived of major side effects and toxicity. Therefore, it is crucial to first establish a clear understanding of the underlying molecular mechanisms through which HDACs can affect fibrotic and/or inflammatory disorders. We presume that, in the following years, novel selective HDACis, such as benzamide and hydrazide inhibitors, could emerge as potential therapeutic options in diseases and disorders other than cancer, leading the way for the development of effective treatments against IPF or inflammatory and autoimmune diseases.

## Figures and Tables

**Table 1:** HDAC inhibitors as anti-fibrotic agents in IPF.

Inhibitor	HDAC Inhibition	Biological Evaluation In Vitro or In Vivo	Anti-Fibrotic Activity	Refs.
RGFP966 (**1**)	HDAC3	Bleomycin-induced pulmonary fibrosis mice	↑ Nrf2 expression, ↑ Inflammatory cytokines	[20,23]
GK444 (**2**), GK718 (**3**)	HDAC1,2,3	Bleomycin-induced pulmonary fibrosis mice	↓ Col1a1 expression, ↓ Fibrotic masses, ↓ Collagen deposition	[25]
J27644 (**4**)	HDAC6,8	TGF-β-treated human pulmonary fibroblasts (HPFs)	↓ Pulmonary fibrosis, ↓ α-SMA, ↓ Col1a1 levels	[28]
Compound **5**	HDAC6			
Compound **6**	HDAC6	Organoid cultures of airway basal cells derived from IPF patients	↓ Proliferation, ↓ Fibrotic sphere formation	[29]
		Ex vivo cultures of human lung tissues	↓ TGF-β dependent fibrogenesis, ↓ Expression of ECM genes	
Compound **7**	HDAC6-TRAIL activation	Bleomycin-induced and silica suspension-induced mice	↓ Proliferation (Fibroblast cell lines NIH/3T3 and HPF)↓ Inflammation, ↓ Collagen deposition	[30]
H10 (**8**)	HDAC6	Bleomycin-induced pulmonary fibrosis mice	↓ TGF-β1-dependent fibrogenesis, ↓ Collagen deposition	[31]
MPT0E028 (**9**)	Pan-HDAC	Human lung fibroblasts	↑ MKP-1, ↓ p38 + ERK phosphorylation, ↓ Smad3 + AP-1 activation, ↓ CTGF expression	[32]
		Bleomycin-induced pulmonary fibrosis mice	↓ Fibrosis score, ↓ Fibronectin, ↓ Collagen, ↓ α-SMA expression	
CUDC-907 (**10**)	Pan-HDAC- PI3K/AKT	Bleomycin-induced pulmonary fibrosis mice	↓ Collagen levels	[33]

↑ Indicates an increase, ↓ indicates a decrease.

**Table 2:** HDAC inhibitors as anti-fibrotic agents in other fibrotic diseases.

Inhibitor	HDAC Inhibition	Biological Evaluation In Vitro or In Vivo	Anti-Fibrotic Activity	Refs.
PCI34051 (**11**)	HDAC8	Isoproterenol-induced cardiac hypertrophy mouse model	↓ Cardiac fibrosis, ↓ TGF-β1/Smad2/3 pathway (Rat cardiac fibroblasts), ↓ Cardiac hypertrophy, ↓ Collagen type I, ↓ Fibronectin, ↓ CTGF	[34,36,41]
		Unilateral ureteral obstruction murine model	↑ Contactin acetylation, ↓ Phosphorylation of Smad3, STAT3, β-catenin, ↓ Snail expression	
Givinostat (**12**)	Pan-HDAC	Mouse model of diastolic dysfunction	↓ Extracellular matrix deposition ↓ Cardiac fibroblast activation	[37,44]
		Mice receiving methionine- and choline-deficient diet	↓ Inflammation, ↓ Hepatic fibrosis	
Rhein (**13**)	Classes I/II HDACs	Hypoxia-treated or TGF-β1-stimulated primary human ventricular cardiac fibroblasts	↓ Collagen contraction, ↑ Smad7 levels, ↑ Smad-specific E3 ubiquitin ligase SMURF2	[38]
CG200745 (**14**)	Pan-HDAC	Col4a3−/− mice, a murine model of Alport syndrome	↓ Kidney fibrosis, ↓ TGF-β-Smad signaling	[39]
TSA (**15**)	Pan-HDAC	Unilateral ureteral obstruction murine model	↓ Interstitial macrophages, ↑ M2c macrophages, ↓ Myofibroblast activation, ↓ Fibrosis	[40]
Vorinostat (**16**)	Pan-HDAC	LX2 cells isolated from human hepatic stellate cells	↓ Cell viability, ↓ Migration, ↓ Colony formation, ↓ Expression of Col1a1, Col3a1, α-SMA and TGF-β genes	[43]
LP340 (**17**)	Class I HDACs	Mouse models of liver fibrosis induced by CCl_4_ treatment or bile duct ligationImmortal human hepatic stellate cells	↓ Liver injury, ↓ Inflammation, ↓ Fibrosis, ↓ miR23a,↓ TGF-β/Smad signaling	[45]

↑ Indicates an increase, ↓ indicates a decrease.

**Table 3:** HDAC inhibitors as anti-inflammatory agents.

Inhibitor	HDAC Inhibition	Biological Evaluation In Vitro or In Vivo	Anti-Inflammatory Activity	Refs.
Compound **19**	Class I and Class II (not HDAC8)	In vitro in murine macrophage J774A.1 cells	↓LDH, blocks NLRP3 inflammasome activation => ↓IL-1β and ↓caspase-1	[60]
SPA3074 (**20**)	HDAC8	In vivo colitis mouse model	↑SOCS1 expression => ↓p-Akt and ↑ERK1/2, ↑p-IκBα, ↓IL-13	[63]
LL87 (**21**)	Class IIa	In vitro in HMDMsIn vivo in rat CIA model	↓IL-1α, ↓MCP-1, ↓GM-CSF, ↓IL-6↓p-Akt, ↓p-ERK1/2	[64]
Compounds **22** and **23**	HDAC6	In vitro in human THP-1 macrophages	↑Acetylation of α-tubulin, ↓LPS-induced *IL1β* mRNA expression, ↓TNF	[70]
Compound **24**	HDAC6	In vitro in murine macrophage J774A.1 cellsIn vivo endotoxic shock mouse model	↓ATP/LPS-induced IL-1β release	[71]
PB131 (**25**)	HDAC6	In vitro in mouse microglia BV2 cellsIn vivo neuroinflammation mouse model	↓IL-10, ↓IFN-γ, ↓IL-1β, ↓IL-2, ↓IL-5, ↑Acetylation of α-tubulin	[72]
Compound **26**	HDAC6	In vivo chronic respiratory infection mouse model	↓IL-1α, ↓IL-1β, ↓IL-4, ↓IL-6, ↓IL-12, ↓IL-17A, ↓IFN-γ	[73]
Compound **27**	HDAC6	In vitro in cutaneous T-cell lymphoma cells and in THP-1 cellsIn vivo in rat AIA model and in mouse CIA model	↑Acetylation of tubulin, ↓TNF-α secretion	[74]
Sodium butyrate (**28**)	Pan-HDAC	In vivo asthmatic mouse model	↓LDH, ↓GATA-3 => ↓IL-5, ↓HIF-1α, ↓VEGF-α, ↓p-PI3K, ↓p-Akt	[77,78,79]
		In vitro in liver macrophages, BMDM and RAW264.7 cells	↓TNF-α, ↓IL-6, ↓Inflammasome activation	
		In vivo atherosclerosis inflammation mouse model	↓TNF-α, ↓IL-1β, ↓IL-6, ↓IL-7A, ↓IFN-γ, ↑IL-10	

↑ Indicates an increase, ↓ indicates a decrease.
